# Damaged Goods

**Published:** 1913-07

**Authors:** 


### "Damaged Goods."

An interesting experiment in the teaching of moral and social lessons is being carried on at the Fulton Theater in New York City. The play which is being presented there to crowded houses is entitled "Damaged Goods." It is the work of a French dramatist, M. Brieux. The thing which marks the production as worthy of serious attention is not the play itself. In fact, it is hardly a play at all. At best it might be called a one-act play with prologue and epilogue. Nor is it the excellence of the performance, although the acting of Richard Bennett and his co-workers is on a high plane of excellence. It is the subject with which the production deals and the serious spirit in which it is dealt with that makes the production a remarkable event. The subject is openly and without circumlocution the terrible character of the disease which attends immorality. It is treated with absolute frankness. Disease is spoken of as a matter of fact just as our grandmothers would have spoken of consumption or typhoid fever or diphtheria. On the other hand, the disease is treated with the same kind of reserve that one would like to see used in referring to leprosy under similar circumstances. In addition, the danger which it is so difficult to avoid in treating of the evils that spring from the sexual relation, the danger of arousing or catering to a prurient curiosity, is skilfully and completely avoided. The lesson which the play aims to teach has several parts. They may be summarized as follows:

1. One of the great scourges of the world, standing alongside of tuberculosis and alcoholism.

2. Its effects are not only personal, they are communicated in even more serious form to wife and children.

3. A father should be as careful in demanding proof of freedom from disease of a suitor for his daughter's hand as in investigating his ability to support her.

4. Society should make it impossible for marriage to take place where the man is tainted with dangerous disease.

5. The great danger is lack of knowledge. In the play the cry of victim after victim, father, wife, the sufferer herself, is, "I didn't know."

The play is, above all, a powerful plea for tearing away the veil of mystery that has so universally shrouded this subject of the penalty of immorality. It is a plea for light on this hidden danger, that fathers and mothers, young men and young women may know the terrible price that must be paid, not only by the generation that violates the law, but by the generations to come. It is a serious question just how the education of men and women, especially young men and young women, in the vital matters of sex relationships should be carried on. One thing is sure, however. The worst possible way is the one which has so often been followed in the past—not to carry it on at all, but to ignore it. Moreover, there is large room for debate as to whether either the novel or the drama is the proper vehicle for such education. There is no question, however, that the author of "Damaged Goods" and the players who are presenting it have set about their mission in the right spirit and have accomplished what they set out to do.—*Outlook.*

---

THEORY AND PRACTICE.—Doctor: As a physician, I must condemn the use of alcoholic beverages. Patient: But you use them yourself. Doctor: Yes; but not as a physician. When I drink, I am nothing but an ordinany human being with a thirst.

